# Paternal Involvement in and Sociodemographic Correlates of Infant and Young Child Feeding in a District in Coastal South India: A Cross-Sectional Study

**DOI:** 10.3389/fpubh.2021.661058

**Published:** 2021-06-04

**Authors:** Prasanna Mithra, Bhaskaran Unnikrishnan, Rekha T, Nithin Kumar, Ramesh Holla, Priya Rathi

**Affiliations:** ^1^Department of Community Medicine, Kasturba Medical College, Mangalore, Manipal Academy of Higher Education, Manipal, India; ^2^Department of Community Medicine, Datta Meghe Institute of Medical Sciences, Wardha, India

**Keywords:** IYCF, involvement, paternal, correlates, India

## Abstract

**Context:** The feeding practices during first 2 years of life determine the overall health and survival during childhood and beyond. Child nutrition is responsibility of both parents and so far emphasis has been laid mostly on mothers. Fathers' involvement toward Infant and Young child feeding (IYCF) has been proved to be of paramount importance and yet it is given limited importance.

**Objectives:** This study aims to study the level of paternal involvement toward IYCF and its associated factors and to assess the knowledge, attitude, and practices toward IYCF in Dakshina Kannada District in South Indian State of Karnataka.

**Settings and Design:** This community-based cross-sectional study was conducted in the coastal District of Dakshina Kannada; Karnataka State in India.

**Methods:** The study included 450 fathers of infant and young children (aged <2 years) in five taluks of Dakshina Kannada district. They were assessed for knowledge, attitude, and practices related to IYCF. Median score for the practice component was considered cut off to classify involvement in IYCF. Data were analyzed using IBM SPSS Statistics for Windows, Version 25.0. Armonk, NY: IBM Corp. Chi-square-test and Binary Logistic Regression with Hosmer-Lemeshow goodness-of-fit model were used. Unadjusted and adjusted odds ratios were generated. A *p*-value of <0.05 was considered statistically significant.

**Results:** Mean age of the study population was 34.6 years (SD, 5.4). The practice scores median (IQR) was 34.0 (IQR, 31.0–39.0), and 40.9% of the participants had poor involvement in IYCF. This was significantly higher among fathers from predominantly urban area. Those who had education above graduation and younger fathers had better involvement in IYCF.

**Conclusions:** Less than half of fathers had poor involvement in IYCF, and it was lower among fathers from urban areas, higher age, and lower educational levels.

## Introduction

Feeding practices of children during infancy and early childhood in the first 2 years of life, determine their overall health status and long-term well-being (1, 2). These practices are collectively referred to as infant and young child feeding (IYCF) practices. They include breast feeding practices, weaning, type and frequency of food items introduced and general care toward the child (2, 3).

Both the parents have responsibility toward nutrition of their child (4). In addition, sociocultural factors play an important role in influencing the decisions related to IYCF at the family and household level (5, 6). Traditionally, responsibility of IYCF rests upon the mothers. Fathers' role is believed to be limited to bread winning for the family. However, when support is received from the father's side, the child receives better nutrition (7–10). Paternal support also reflects the extent to which they have maintained a balance between their work and family life (11).

However, fathers are not involved in child-feeding research and policies (12, 13). There have been several strategies implemented to support individual mothers (10, 14). Many times fathers of infants and young children would have the right attitude, but that may not translate to adequate involvement in IYCF (2).

In addition, there is a lack of evidence regarding the level of paternal involvement in infant-feeding practices and its associated factors, especially in the Southern part of India (3, 12, 15–17). This study was carried out to seek an insight about the level and determinants of paternal involvement in their IYCF, their knowledge, attitude, and practices related to IYCF in Dakshina Kannada District of Karnataka State.

## Methods

This community-based cross-sectional study was carried out in Dakshina Kannada, a coastal District in Karnataka State, among select fathers of infants (children aged < 1 year) and young children [children aged 12–23 months (18)], between March 2019 and February 2020. Sample size was calculated with an assumption of 50% of the fathers with poor involvement in infant and young child feeding, 5% absolute precision, 95% confidence interval, and adding 20% non-response error, as 450, using the following formula:

*n* = *z*^2^*pq*/*d*^2^*z* = 1.96, *p* = 0.5, *q* = 1-*p* and *d* at 5%

Approval was obtained from the Institutional Ethics Committee of study Institute, and necessary permissions were taken from the Head of the Institute and District Health Officer (Dakshina Kannada). Two Research Assistants were recruited for data collection, from the social work background with good communication skills, after competitive interview process. To select the study participants, stratified multistage random sampling technique was used; the level of stratification being at taluk (subdistrict) level. At first, a list of community health centers (CHC) and primary health centers (PHC) was obtained and at each of the five taluks in Dakshina Kannada. For the community, a PHC is the first level of contact with a medical doctor. The next level of health care for the community is CHC; with the availability of specialist health care services. Health centers were selected using simple random sampling, using probability proportionate to size technique (to decide the number of fathers to be included from area belonging to each center), based on the population covered by them. One ward (administrative unit area) was selected using simple random sampling, among the area covered by the health center. A list of infants and young children was obtained for these wards and further, the households with eligible children were selected till the required sample size was met. Those selected fathers were visited in their houses and their identity was confirmed. When they were not present in their houses, they were contacted through the family members and visit was paid on a mutually convenient time.

The fathers were approached and explained about the objectives of the study in a language they understood. A written informed consent was taken from each of them. Data were collected using a pretested, validated semistructured proforma in the local language (Kannada). The proforma included general information of the subjects, family details, knowledge, attitude, and involvement in IYCF and other practices related to IYCF. The responses of knowledge, attitude, and involvement were in the form of 5-point Likert scale, wherein a score of 5 meant they strongly agreed to the statement and 1 was the score for strongly disagree. The attitude and practices related to IYCF were assessed for the youngest child among the fathers with more than one child.

Collected data were coded and analyzed using IBM SPSS Statistics for Windows, Version 25.0. Armonk, NY: IBM Corp. Results were expressed as proportions, mean (with standard deviation) using appropriate tables. For the scores for practice (involvement) of IYCF, total was calculated and the median score was set as the cut-off score for classifying the poor involvement. For factors associated with fathers' involvement in IYCF, Chi square-test, and Binary Logistic Regression analysis were used. Adjusted and unadjusted odds ratios were calculated with 95% confidence intervals. The fit of the logistic model was assessed with the Hosmer and Lemeshow goodness-of-fit test. A *p*-value < 0.05 was considered statistically significant.

## Results

This study included a total of 450 participants, with mean age of 34.6 years (SD: 5.4). There were 90 subjects from each taluk of Dakshina Kannada District (Bantwal, Belthangady, Mangalore, Puttur, and Sullia). The demographic profile of the study population is described in Table 1. Among the participants, 64.2% were in the age group of 31–40 years and 44.4% were educated up to high school. When their individual occupations were regrouped, more than half of the participants were in the combined group of unskilled and semiskilled types of work. Overall, 43.8% of the participants were first-time fathers.

**Table 1:** Demographic characteristics of the study population (*N* = 450).

**Characteristics**		**No. (%)**
Age group (years)	≤ 30	112 (24.9)
	31–40	289 (64.2)
	≥41	049 (10.9)
Educational status	<High school	200 (44.4)
	Completed high school	115 (25.6)
	Pre-degree	055 (12.2)
	≥Graduation	080 (17.8)
Occupation	Unskilled	132 (29.3)
	Semiskilled	111 (24.7)
	Skilled	145 (32.2)
	Professional	062 (13.8)
Parenthood	First-time fathers	197 (43.8)
	Experienced fathers	253 (56.2)
Locality	Predominantly urban	090 (20.0)
	Predominantly rural	360 (80.0)
	Total	450 (100)

Table 2 describes the knowledge and perceptions scores of participants on IYCF. They showed positive responses to being aware about the Anganwadi facilities, importance of breast feeding for baby's overall health and growth, perception of being in a position to identify if baby is hungry, and feeling that baby is getting enough nutrition. They also had high awareness score toward the duration of breastfeeding being important aspect of child nutrition (4.34, SD: 0.50). However, other aspects of knowledge and perceptions, the average scores were less than the midline score of 3. With respect to the child's nutrition, the participants felt it was mostly the mother's responsibility rather than paternal.

**Table 2:** Knowledge and perceptions related to IYCF among the study participants (*N* = 450).

**Characteristics**	**Mean (SD)**
Breastfeeding is very important for baby's overall nutrition	4.79 (0.46)
Duration of breastfeeding is one of the important aspects of child nutrition	4.34 (0.50)
Mother has the most important role to look after the baby/child and his/her food items^#^	1.89 (0.75)
Father should be involved in deciding when to start weaning	2.98 (0.86)
Father should be involved in deciding which food items to be given to child while initiating complementary feeding	2.76 (0.84)
Age-wise new food items have to be introduced into child's diet	2.84 (0.97)
Father should have expertise in deciding the diet of the baby/child	2.47 (0.98)
Own baby/child consumes the food in right quantities	4.10 (0.61)
Own baby is being given balanced diet^*^	3.33 (0.88)
Can recognize whenever own baby/child is hungry	3.67 (0.84)
Own child is being provided adequate nutritional supplements	2.96 (0.90)
Father should have regular discussions with the mother of the baby/child regarding the food items being given	2.81 (0.92)
Better nutrition of child/baby depends upon joint decision of both the parents	2.88 (0.96)
Child would eat better in the presence of father	2.86 (0.93)
Your involvement in feeding of your baby/child makes your wife more comfortable	2.86 (0.96)
Pleasure in taking care of own baby/child's nutrition	2.90 (0.97)
Aware of the immunization schedule applicable for the baby/child	3.01 (1.03)
In addition to the diet, it is important to comply with the immunization schedule applicable for the baby/child	2.01 (1.27)
Having a feel that adequate involvement in own baby/child nutrition	3.28 (1.38)
Aware of nearest Anganwadi center and its services toward child nutrition	4.79 (0.46)

*The Likert score pattern: strongly agree (5), agree (4), neutral (3), disagree (2), strongly disagree (1).*

* *Balanced diet includes all required nutrients to your baby/child in correct amounts.*

# *Scores reversed and added up to reflect higher awareness with higher scores*.

Overall, the participants had favorable attitude toward the child nutrition and their involvement. The scores for readiness to adapt to changes in feeding habits and to accept advises given by the doctors of other health care providers were high. In addition, they had positive scores for listening to spouse suggestions regarding feeding practices of the baby and to further improve their involvement in IYCF. The mean (SD) scores of the attitude among participants to enhance involvement in IYCF is given in Table 3.

**Table 3:** Attitude of the study participants toward enhanced involvement in IYCF (*N* = 450).

**Characteristics**	**Mean (SD)**
Willing to adapt to changes needed in baby/child's feeding	4.16 (0.89)
Willing to enhance involvement in baby/child's feeding	3.93 (0.73)
Readily listen to the suggestions made by spouse regarding baby/child's feeding	4.03 (0.76)
Ready to accept the suggestions made by the doctor/health worker	4.14 (0.77)

The scores of the practices in IYCF are described in Table 4. The scores were highest for accompanying the spouse to hospital during illness, followed by purchasing food items and playing with the child. For the remaining items, the scores were around the middle score of 3. Also preparing the food items by themselves or assisting the wife to prepare food for child and feeding the child on own motivation were the least preferred practices by study participants. The practice scores were added up and median (IQR) was calculated as 34.0 (IQR, 31.0–39.0). All the participant fathers whose scores were below the level of median were categorized as having poor involvement in IYCF practices. In total, 40.9% of the fathers had poor involvement in IYCF based on the median cut off of practice scores.

**Table 4:** Practices of the study participants related to IYCF (*N* = 450).

**Practices**	**Mean (SD)**
Seeking advice on baby's/child's diet from doctor/health worker	2.03 (1.43)
Have taken the baby/child for growth monitoring	1.98 (1.35)
Have the growth chart updated by health worker/doctor	3.94 (1.10)
Prepare/assist the spouse to prepare food items for baby/child	1.19 (0.63)
Feeding the child by self	1.65 (1.19)
Give the food items procured from Anganwadi	1.78 (1.38)
Wash or help the spouse wash/clean the utensils used for baby/child feeding	1.83 (1.38)
Accompany wife to buy vegetable and other food items for baby/child	3.84 (1.58)
Assist the wife in cleaning/bathing the baby/child	2.34 (1.60)
Accompany wife during her/baby's health check ups	4.26 (1.24)
Play with baby/child during leisure time	3.56 (1.35)
Take the baby/child outside the house for playing	2.98 (1.54)
Changing or helping the wife change the diapers/nappies of baby/child	1.66 (1.09)
Take care of baby when wife is not around	1.81 (1.29)

In addition, for their diet of the child/baby, 69.8% of the participants and their families have been giving rice-based items. In total, 369 (82.0%) participants said breast feeding was continued beyond the weaning time of 6 months of age. Cow's milk was being given to babies/children of 159 (35.3%) participants. Less than 15% of the children of participants received complementary food purchased from outside. Other food items given to their babies/children were mashed fruits and vegetables, biscuits, fruit juices, goat milk, bread rusk, beaten rice, etc.

The univariate analyses of the factors influencing the practice of involvement in IYCF are depicted in Table 5. With increasing age, the level of poor involvement was higher (36.6% in <30 years age group vs. 46.9% in participants aged 41 years and above). However, this trend was not found to be statistically significant (*p* = 0.119). The involvement was found to be better among the participants who were educated above graduation (66.2%). The levels of poor involvement were higher among those with education high-school and lesser. However, those with pre-graduation-level education had highest level of poor involvement (50.9%). This difference across the educational status was not found to be statistically significant (*p* = 0.264). Occupational status showed an extreme of findings. Those who were unskilled had better involvement than semiskilled and skilled workers. However, the involvement was highest among those who were having professional jobs. We did not find this difference statistically significant (*p* = 0.062).

**Table 5:** Univariate analysis of the sociodemographic correlates of poor involvement in IYCF among study participants (*N* = 45).

**Characteristics**		**IYCF involvement**		***p*-value**
		**Good**	**Poor**	
		**No. (%)**	**No. (%)**	
Age group (years)	≤ 30 (*n* = 112)	071 (63.4)	041 (36.6)	0.119
	31–40 (*n* = 289)	169 (58.5)	120 (41.5)	
	≤ 41 (*n* = 49)	026 (53.1)	023 (46.9)	
Educational status	Less than high school (*n* = 200)	118 (59.0)	082 (41.0)	0.264
	Completed high school (*n* = 115)	068 (59.1)	047 (40.9)	
	Pre-degree (*n* = 55)	027 (49.1)	028 (50.9)	
	Graduation and above (*n* = 80)	053 (66.2)	027 (33.8)	
Occupational status	Unskilled (*n* = 132)	083 (62.9)	049 (37.1)	0.062
	Semiskilled (*n* = 111)	063 (56.8)	048 (43.2)	
	Skilled (*n* = 145)	076 (52.4)	069 (47.6)	
	Professional (*n* = 62)	044 (71.0)	018 (29.0)	
Locality	Predominantly urban (*n* = 90)	016 (17.8)	074 (82.2)	<0.0001^*^
	Predominantly rural (*n* = 360)	250 (69.4)	110 (30.6)	
Parenthood	First-time fathers (*n* = 197)	126 (64.0)	071 (36.0)	0.065
	Experienced fathers (*n* = 253)	140 (55.3)	113 (44.7)	
Gender of the youngest child	Male (*n* = 222)	125 (56.3)	97 (43.7)	0.23
	Female (*n* = 228)	141 (61.8)	87 (38.2)	
Total		266 (59.1)	184 (40.9)	

* *p value is significant at 0.05 level*.

The study participants residing in predominantly urban locations had higher levels of poor involvement in IYCF as compared with those residing in predominantly rural locations (82.2 vs. 30.6%). This difference was found to be statistically significant (*p* < 0.0001). The participants who became fathers for the first time had better involvement than the experienced fathers (64.0 vs. 55.3%). This difference was not found to be statistically significant (*p* = 065). In addition, among fathers who had female children, poor involvement was 38.2% and among those with male children, it was noted to be 43.7%. This difference was not found to be statistically significant.

Table 6 describes the multivariate analysis of the factors determining the poor paternal involvement in IYCF. The findings were similar to univariate analyses. Outcome of poor involvement was adjusted for age group, education, occupation, locality of stay, gender of the child, and parenthood status. The analysis model revealed a significant association between poor involvement and locality of stay, status of parenthood (first time vs. experienced fathers), occupational status, and gender of the child. Those staying in predominantly urban areas were 16.83 times (95% CI, 8.46–33.49) more likely to be having poor involvement as compared with those from predominantly rural areas. In addition, participants with unskilled, skilled, and semiskilled occupations differed significantly from those in professional jobs. Those fathers with male children showed significantly higher level of poor involvement than those with female children (OR, 1.79; 95% CI, 1.11–2.90). Age of the youngest child did not have any effect in paternal involvement in IYCF (OR, 0.99; 95% CI, 0.96–1.02).

**Table 6:** Multivariate analysis of covariates of poor involvement of study participants in IYCF (*N* = 450).

**Characteristics**		**Unadjusted odds ratio** **(OR, 95% CI)**	***p-*value**	**Adjusted OR** **(95% CI)**	***p-*value**
Age group (years)	≤ 30	–		–	
	31–40	1.23 (0.78–1.93)	0.37	1.33 (0.79–2.25)	0.28
	≥41	1.53 (0.78–3.02)	0.22	1.51 (0.67–3.41)	0.32
Educational status	Less than high school	1.36 (0.79–2.35)	0.26	1.17 (0.58–2.36)	0.67
	Completed high school	1.36 (0.75–2.46)	0.31	0.88 (0.43–1.80)	0.72
	Pre-degree	2.04 (1.01–4.11)	0.05^*^	1.99 (0.88–4.50)	0.10
	Graduation and above	–		–	
Occupation grade	Unskilled	1.44 (0.75–2.77)	0.27	2.48 (1.04–5.89)	0.04^*^
	Semiskilled	1.86 (0.96–3.62)	0.07	2.61 (1.09–5.45)	0.02^*^
	Skilled	2.22 (1.17–4.20)	0.01^*^	2.44 (1.05–5.2)	0.03^*^
	Professional	–		–	
Residence locality	Predominantly rural	–		–	
	Predominantly urban	10.51 (5.86–18.87)	<0.0001^*^	16.83 (8.46–33.49)	<0.0001^*^
Parenthood status	First-time fathers	–		–	
	Experienced fathers	1.43 (0.98–2.09)	0.07	1.34 (0.84–2.13)	0.21
Gender of the youngest child	Male	1.26 (0.86–1.83)	0.23	1.79 (1.11–2.90)	0.02^*^
	Female	–		–	
Age of the youngest child (months)		0.99 (0.96–1.01)	0.32	0.99 (0.96–1.02)	0.63

* *p-value significant at 0.05 level*.

Overall, 67.6% of them utilized the Anganwadi services available to them. In total, 263 (58.4%) of the fathers felt they need an organized and regular information sharing program or a center on IYCF, which could further enhance their involvement and optimize the related practices.

## Discussion

Our study included 450 fathers with children aged <2 years in the Dakshina Kannada District. Overall poor involvement in IYCF was seen among 40.9% of the fathers. Usually, fathers involve minimally in feeding practices and related decision making during the earlier months of their child. The fathers' care toward child feeding becomes active beyond the age of 2 years and hence the needed care for young child may not be adequate (10). Across different regions of the world, there are studies which reported that there was limited and also varied involvement of fathers in their young child's feeding and rearing. The study by Mallan et al. among Australian fathers reported paternal involvement of 42% in child feeding (19) and Abera et al. from Ethiopia in 2017 reported 72.4% paternal involvement in breastfeeding practices (5). Tikotzky et al. in Israel assessed the involvement of fathers and mothers in overall and nighttime infant caregiving, and they observed that mothers' involvement was significantly higher than that of fathers in overall baby care (20).

A review of existing evidence by Neha Khandpur et al. in 2014 reported the scantiness of studies and validated paternal involvement assessment techniques (12). They also noted that, due to changes in norms of society, fathers' involvement in child care has increased as compared with the level a few years ago (12). The existing evidence also depicts that paternal involvement in IYCF is limited and is influenced by several region and culture-specific factors (7, 11, 21, 22). A study by Inbaraj et al. in Bangalore in 2020 noted that religion, type of family, and per capita income were independently associated with poor involvement of fathers in child feeding. In their study, paternal feeding practices did not contribute to child's nutritional status (4). Ito et al. in Japan reported that paternal infant care was inversely associated with breastfeeding during the first 6 months of life (7).

The awareness and attitude domains in our study showed positive patterns. They had awareness about key aspects of child feeding and overall care, added by their favorable attitudes to be increasingly involved in feeding of their child, care during illness, etc. A review and concept analysis by Nigel Sherriff et al. focused on breastfeeding practices and positive paternal attitudes toward it especially during the post-partum period and early infancy of their child (23).

Our study noted that with increasing age, the level of paternal involvement in IYCF decreased. First-time fathers had better involvement than the fathers with more than one child. This could reflect the fatigue which sets in with progressing time. In addition, in our study, the involvement was found to be better among the participants who were educated above graduation.

Occupational status in our study showed a mixed result that those who were skilled had better involvement than semiskilled and unskilled workers, and the involvement was highest among those who were having professional jobs. This shows the influence of demographic factors on the pattern of paternal care. The same has been documented by previous studies. In the study by Abera et al. it was associated with several factors like educational status, monogamy matrimonial status, visiting health facility, having wives with positive perception about fathers' involvement, and having access to media source at home (5). In the study by Mallan et al. work place time adversely affected the frequency of fathers eating meals with their child (19). Shefaly Shorey et al. in their longitudinal study on paternal involvement at 6 months of post-partum reported that fathers (first-time and experienced) who were involved during their infant's birth were also involved at 6-month post-partum (16). In our study, we also noted that the level of involvement was significantly higher among fathers with female children. This could be due to higher literacy rates in the study area and family characteristics. However, in a study by Blissett et al. there was no gender difference with respect to paternal feeding practices (24).

This study targeted the relatively neglected area of paternal practices and used a self-developed tool to measure the involvement. In addition, it included a representative population spread across the district. This could also further be replicated in other areas and if required, with suitable modifications. However, a limitation of this study could be that, focus was maintained toward fathers, and details of the mothers' education and awareness were not measured, which could be an influencing factor in the paternal IYCF practices.

Apart from the level and sociodemographic determinants of paternal involvement in IYCF, this study also points toward a non-availability of uniform tool to assess the same. Considering the less attention given to fathers' involvement in IYCF, there is a need to enhance the same to ensure best feeding and caring practices for infants and young children. Further interventions targeting the fathers on IYCF could be planned. This in turn would benefit the practitioners and policy makers in enhancing the paternal involvement in IYCF and planning the interventions accordingly.

## Conclusions

Close to half of fathers of children aged 2 years or less showed poor involvement in IYCF in the District of Dakshina Kannada, despite having adequate knowledge and favorable attitude. Paternal involvement was influenced by the locality of residence (predominantly urban being more with poor involvement). Those who had education above graduation had better involvement than others. Occupational status did not have any association with involvement in IYCF.

## Data Availability Statement

The datasets presented in this study can be found in online repositories. The names of the repository/repositories and accession number(s) can be found here: doi:10.17605/OSF.IO/K9YXT

## Ethics Statement

The studies involving human participants were reviewed and approved by Institutional Ethics Committee of Kasturba Medical College, Mangalore, India. The patients/participants provided their written informed consent to participate in this study.

## Author Contributions

PM and BU conceptualized this research. PM, RT, and NK developed the methodology. PM and NK analyzed the data and interpreted the results. BU, RH, and PR critically appraised the interpretations. All the authors contributed to the writing of this manuscript.

## Conflict of Interest

The authors declare that the research was conducted in the absence of any commercial or financial relationships that could be construed as a potential conflict of interest.
